# Resources needed by critical access hospitals to address identified infection prevention and control program gaps

**DOI:** 10.1017/ash.2024.32

**Published:** 2024-03-15

**Authors:** Mounica Soma, Jody Scebold, Angela Vasa, Teresa Ann Fitzgerald, Kate Tyner, Satya Kumar Lalam, Sue Beach, Muhammad Salman Ashraf

**Affiliations:** 1 National Infection Control and Strengthening Collaborative, Nebraska Medicine, Omaha, NE, USA; 2 Nebraska Infection Control Assessment and Promotion Program, Nebraska Medicine, Omaha, NE, USA; 3 Biomedical Informatics, University of Nebraska Medical Center, Omaha, NE, USA; 4 Healthcare Associated Infections and Antimicrobial Resistance Program, Division of Public Health, Nebraska Department of Health and Human Services, Lincoln, NE, USA; 5 Division of Infectious Diseases, University of Nebraska Medical Center, Omaha, NE, USA; 6 Global Center for Health Security, University of Nebraska Medical Center, Omaha, NE, USA

## Abstract

**Objective:**

The study examined resources needed by Infection Preventionists (IP) to address infection prevention and control (IPC) program gaps.

**Design:**

A 49-question survey.

**Setting:**

Licensed Critical Access Hospitals (CAHs) in Federal Emergency Management Area (FEMA) Region VII.

**Participants:**

IP at licensed CAHs.

**Methods:**

The survey conducted between December 2020 and January 2021 consisted of questions focusing on four categories including IPC program infrastructure, competency-based training, audit and feedback, and identification of high-risk pathogens/serious communicable diseases (HRP/SCD). An IPC score was calculated for each facility by totaling “Yes” responses (which indicate best practices) to 49 main survey questions. Follow-up questions explored the resources needed by the CAHs to implement or further strengthen best practices and mitigate IPC practice gaps. Welch t-test was used to study differences in IPC practice scores between states.

**Results:**

50 of 259 (19.3%) CAHs participated in the survey with 37 (14.3%) answering all 49 questions. CAHs responding to all questions had a median IPC score of 35. There was no significant difference between IPC practice scores of CAHs in NE and IA. The top three IPC gaps were absence of drug diversion program (77%), lack of audits and feedback for insertion and maintenance of central venous catheters (76%), and missing laboratory risk assessments to identify tests that can be offered safely for patients under investigation for HRP/SCD (76%). Standardized audit tools, educational resources, and staff training materials were cited as much-needed resources.

**Conclusion:**

IPC practice gaps exist in CAHs. Various resources are needed for gap mitigation.

## Introduction

Critical Access Hospitals (CAHs) are healthcare facilities that meet specific criteria outlined by Centers for Medicare and Medicaid Services (CMS). These criteria include requirements for CAHs to be located in rural area or an area that is treated as rural in states with established State Medicare Rural Hospital Flexibility Program along with being designated by the state as a CAH. They must be located at least 35 miles away from other hospitals (or more than 15 miles in areas with mountainous terrain or only secondary roads; or were certified as a CAH based on state designation as a “necessary provider” of health care services to residents in the area prior to January 1, 2006). In addition, have no more than 25 inpatient beds, maintain no less than 96 hours average length of stay annually, provide 24/7 emergency care, and be Medicare-certified. CAHs play a crucial role in serving rural and underserved communities across the nation by addressing their unique healthcare needs.^1^ They face similar challenges related to limited resources, staffing shortage, and geographic isolation impacting access to subject matter experts and educational opportunities.^2–4^ Furthermore, confidentiality and privacy concerns in close-knit communities may also limit CAHs from sharing their program struggles with others.^5^ These challenges have the potential to adversely impact infection prevention and control (IPC) programs in CAHs.^2–5^

CAHs are required by the CMS to have an IPC program that adheres to nationally recognized principles.^6^ Essential elements of an IPC program are designed to prevent the spread of infection in a variety of healthcare settings.^7–10^ IPC program activities are prioritized through the development of program goals and measurable outcomes after conducting an annual facility IPC risk assessment.^11,12^ An annual risk assessment can identify gaps in an IPC program. A gap is defined as variation in IPC program resources, infrastructure, or processes from the established evidence-based guidelines or institutionally defined best practices.^13^ Identifying and mitigating IPC program gaps can lead to improved patient outcomes.^14^ Making implementation tools and other practical resources widely available can assist IPC programs in their efforts to follow evidence-based guidelines and mitigate IPC program gaps.^15,16^ However, there is a paucity of studies evaluating the resources needed by infection preventionists and other IPC program leaders in CAHs to mitigate IPC gaps. This study focuses on identifying the resources and tools needed to address IPC gaps in CAHs.

## Methods

### Study design and setting

The needs assessment survey was developed leveraging unpublished data from IPC program assessments conducted by Nebraska Infection Control Assessment and Promotion Program (ICAP). ICAP is funded by the Nebraska Department of Health and Human Services Healthcare-Associated Infection and Antimicrobial Resistance (HAI/AR) program through a Centers for Disease Control and Prevention (CDC) grant to assist healthcare facilities in improving their IPC programs. From 2015 to 2017, 36 acute care hospital IPC programs in Nebraska were assessed by ICAP using standard CDC and CMS survey tools.^7,17^ The data gathered by ICAP provided insight into which IPC areas required additional focus in the needs assessment survey. For example, lack of competency-based training programs and failure to perform audits and feedback appeared to be a recurrent theme in several IPC domains. In addition, in-depth assessments related to identifying and isolating patients with potential high-risk pathogens or serious communicable diseases were lacking in the previous assessments. Therefore, the needs assessment survey explored these IPC program areas along with general infrastructure in further detail. The focus of the needs assessment survey was different from prior work during 2015–2017, which identified IPC gaps in Nebraska hospitals but did not systematically investigate the resources needed by the hospitals to mitigate those gaps. It required development of a new online survey tool using the Research Electronic Data Capture (REDCap) platform (Supplementary Appendix 1 (online)).^18,19^ The targeted facilities for this survey included CAHs in Federal Emergency Management Area Region VII, which includes Missouri, Kansas, Iowa, and Nebraska.

The 49-question needs assessment survey consisted of categories focusing on (1) IPC program infrastructure (*n* = 13 questions), (2) Competency-based training (*n* = 11 questions), (3) Audit and feedback (*n* = 11 questions), and (4) Identification and isolation of high-risk pathogens/serious communicable diseases (*n* = 14 questions). In addition to the 49 items, the survey also included two identifier fields, facility name and primary contact information (name, email address, and phone number of the individual completing the survey). Each needs assessment question was a single answer choice type with three options, “Yes,” “No,” and “Not Sure.” The expected answer for every question was a “Yes” indicating best practice recommendations. Respondents who selected “Yes” were asked to identify specific resources (from a list of pre-identified resources) that would assist in further improving their existing practices. Respondents who answered “No” (indicating an IPC gap) were asked to identify specific resources that would assist in mitigating the identified IPC gap. “Yes” and “No” options also had a free text field for the respondents to enter if the resource did not exist in the list provided.

### Selection and description of participants

As of November 2020, there were 259 licensed CAHs in FEMA Region VII (NE- 64; MO-31; KS- 83; IA- 81). Most CAHs in all four states were located in rural areas (NE-68.8%; IA-50.6%, MO-51.4%, KS-84.1%). The median bed size for CAHs in each of the states was 25 beds (NE range 10–25, IA range 13–25, MO range 3–25, KS range 10–25). The target audience was CAH infection preventionists or individuals with multiple responsibilities within the CAH to include infection prevention activities. Outreach to the target audience was done with the assistance of multiple partners including state HAI/AR Program Coordinators, state/local Association for Professionals in Infection Control and Epidemiology chapters, state hospital associations, and/or individual hospital systems. Partner organizations were given the opportunity to distribute the invitation and survey link to infection preventionists within their states or share their contact information for direct recruitment by the study team. The survey was left open for two months of data collection and participants were sent a total of three follow-up email reminders. There were no financial or other incentives to complete the survey. The survey was closed on January 21, 2021, and all responses were collected into the REDCap database.

### Data analysis

Surveys with a response to at least one question in any of the four categories were included in data analyses. Surveys that did not include identifying information were excluded from the analyses. Duplicate surveys were received from 3 CAHs with same individuals submitting the initial and subsequent surveys for each of the facilities. Therefore, only the most recent surveys from these 3 CAHs were included. One acute care hospital in Nebraska that was not licensed as CAH was excluded from the study.

An IPC practice score was calculated to identify IPC gaps. The score was calculated for each CAH by totaling “Yes” responses. A “No” or “Not Sure” response was both counted as an IPC gap. The participating CAH was assigned a score of 1 for each question answered “Yes.” The maximum possible score was 49. Specific IPC practice gaps and categories with the highest percentage of “No” and “Not Sure” responses were identified to be of highest need for the facilities.

Responses from all participants were de-identified for review. Descriptive analyses of responses from all 50 CAHs were performed to evaluate frequency of gaps and most requested resources. Welch t-test was used for statistical analysis to study differences in IPC practice score of hospitals between the two states from which multiple facilities completed assessments. Only those CAHs that answered all 49 questions were included in the Welch *t*-test analysis. A *P*-value <.05 was considered statistically significant. Wilcoxon-Mann-Whitney *U* test was used to compare bed size of responders vs. non-responders in NE and IA. Furthermore, we studied the difference in response rates of CAHs located in urban versus rural areas of NE and IA using χ^2^ test of independence. Microsoft Excel, R statistical software, and Statistical Analytical Software were used for data analyses. The data for the CAH bed sizes and urban vs rural designation were gathered from publicly available datasets.^20,21^

## Results

The needs assessment overall survey response rate was 19.3% (50 of 259 CAHs) with large variations among four states. Nebraska had the highest response rate with 33 CAHs (51.6%) responding to the survey, followed by 16 (19.8%) in Iowa, 1 (1.2%) in Kansas, and none (0%) in Missouri. Most facilities (n = 37; 23 in NE, 14 in IA) answered all 49 questions and 13 (10 in NE, 2 in IA, 1 in KS) partially completed the survey, answering a median of 24 questions (range 13 to 42). A total of 85 CAHs were in rural areas of NE and IA and 60 were located in urban areas. Even though CAHs in rural areas of NE and IA appeared to have higher response rate as compared to the CAHs in urban areas, the difference was not statistically significant. (40% versus 25% respectively, *P*-value .060). Furthermore, bed sizes of responding facilities were also similar to non-responding facilities (median bed size 25 [range 10–25] for responders vs. 25, [range 13–25] for non-responders; *P*-value .074). While the median IPC score for all 50 facilities included in the descriptive analyses was 30.5 (range 5–48), the median IPC score was 35 (range 13–48) for 37 facilities that completed entire survey. There was no significant difference between IPC practice scores of 23 CAHs in NE and 14 in IA completing the entire survey (average score 33.17 vs 36.14; *P*-value .296).

**Figure 1.**
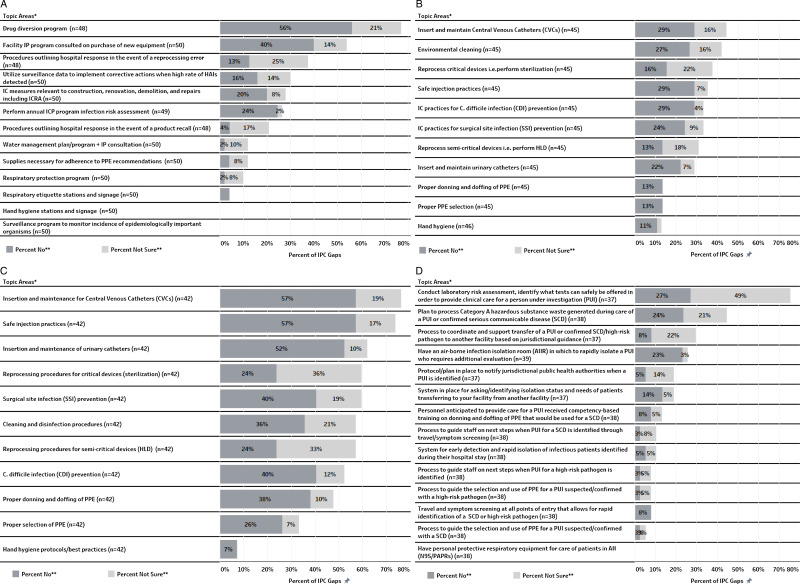
(A) Frequency of IPC Gaps Related to Infection Control Program and Infrastructure. *Note*: The overall gap percent for the category is 21.40%, IPC, Infection Prevention and Control. (B) Frequency of IPC Gaps Related to Competency-Based Training. *Note*: The overall gap percent for the category is 24.18%, IPC, Infection Prevention and Control. (C) Frequency of IPC Gaps Related to Audit and Feedback. *Note*: The overall gap percent for the category is 40.84%, IPC, Infection Prevention and Control. (D) Frequency of IPC Gaps related to serious communicable diseases/high-risk pathogens. *Note*: The overall gap percent for the category is 13.43%, IPC, Infection Prevention and Control. *Topic areas represent the questions asked on the survey; “n” represents count of all yes, no, and Not Sure responses combined for each IPC practice. **The graphs include only the “No” and “Not Sure” response data of the participating facilities. A “No” response indicates that the facility doesn’t have the specific procedure/process/program currently in place while a “Not Sure” response indicates the facility either doesn’t have or is unsure of having a specific procedure/process/program in place.

The two categories with the highest IPC practice gaps were Audit and Feedback and Competency-Based Training with 40% and 24% of the responses marked as “No” or “Not Sure” (Figure 1(A–D)). The most commonly identified IPC practice gaps included absence of a drug diversion program (77%; 37 of 48 respondents), lack of audit and feedback for insertion and maintenance for central venous catheters (76%; 32 of 42), failure to conduct a risk assessment for the laboratory identifying what tests can be safely offered to provide appropriate clinical care for a person under investigation for serious communicable disease (76%; 28 of 37) and lack of audits and feedback for safe injection practices (74%; 31 of 42). Table 1 describes all the IPC practice gaps that were identified in the majority (>50%) of the CAHs responding to various IPC practice questions along with the most commonly mentioned resources needed to mitigate those gaps. Most CAHs cited a standardized audit tool, educational resources, and staff training materials as much-needed resources (Table 1).

**Table 1.** Resources requested for the identified Infection Prevention and Control Gaps (>50% “No” and “Not Sure”)

Identified gap^a^ (gap frequency)	*n* (no. of respondents)	Percent (%)	Top 3 requested resources^b^
Absence of a drug diversion program	48	77%	• A policy/protocol template inclusive of steps to follow in an investigation of drug tampering • An educational resource to train personnel on drug diversion • A guide for creating and implementing a drug diversion program
Lack of audits and feedback for insertion and maintenance of Central Venous Catheters (CVCs)	42	76%	• Standardized CVC insertion and maintenance audit tool (template or mobile app to assist audits) • Educational resources to train personnel on how to provide and receive feedback • Educational resources to train personnel to perform audits
Failure to conduct a risk assessment for the laboratory, identifying what tests can safely be offered to provide appropriate clinical care for a Person Under Investigation	37	76%	• Risk Assessment Template • Mitigation toolkit • Stable workforce (e.g. mitigation strategies for staff turnover) • Access to ongoing equipment readiness guidance
Lack of audits and feedback for safe injection practices	42	74%	• Standardized safe injection practices audit tool (template or mobile app to assist audits) • Educational resources to train personnel on how to provide and receive feedback • Educational resources to train personnel to perform audits
Lack of audits and feedback for insertion and maintenance of (indwelling) urinary catheters	42	62%	• Standardized urinary catheter insertion and maintenance audit tool • Educational resources to train personnel on how to provide and receive feedback • Educational resources to train personnel to perform audits
Lack of audits and feedback on adherence to reprocessing procedures for critical devices	42	60%	• Standardized audit tool for reprocessing critical devices (template or mobile app to assist audits) • Educational resources to train personnel on how to provide and receive feedback • Educational resources to train personnel on how to receive feedback • Educational resources to train personnel to perform audits
Lack of audits and feedback on adherence to recommended IC practices for Surgical Site Infection (SSI) prevention	42	60%	• Standardized audit tool for IP practices related to SSI prevention • Educational resources to train personnel on how to provide and receive feedback • Dedicated FTE for performing audits
Lack of audits and feedback on adherence to cleaning and disinfection procedures	42	57%	• Standardized cleaning and disinfection procedure audit tool • Educational resources to train personnel on how to perform audits and provide and receive feedback • Dedicated FTE for performing audits
Lack of audits and feedback on adherence to reprocessing procedures for semi-critical devices	42	57%	• Standardized audit tool for reprocessing semi-critical devices • Dedicated FTE for performing audits • A tool or database for storing audit and feedback data
Facility procedures lacking consultation with the Infection Prevention program upon purchase of new equipment or products	50	54%	• A procedure template for new products/purchases that incorporates IP program consultation • Focus of regulatory authorities during surveys • Leadership buy-in
Lack of audits and feedback on adherence to recommended infection control practices for *Clostridioides difficile infection* (CDI) prevention	42	52%	• Standardized audit tool for IP practices related to CDI prevention • Educational resources to train personnel on how to perform audits and provide and receive feedback • Dedicated FTE for performing audits

Note. FTE, full-time equivalent; IP, infection prevention; IC, infection control.

a Identified gaps refer to those infection prevention and control practices that were marked “No” & “Not Sure” by >50% of the respondents.

b The top 3 requested resources are based on the responses provided by CAHs that do not have a procedure/process in place.

Other notable gaps included lack of annual IPC program risk assessment and lack of implementation of infection control measures relevant to construction, renovation, demolition, and repairs in over a quarter of CAH (27%; 13 of 49 and 28%; 14 of 50, respectively). Moreover, greater than one-third of CAHs did not implement competency-based training related to safe injection practices and insertion and maintenance of central venous lines (36%; 16 of 45 and 44%; 20 of 45, respectively). When looking into preparation for serious communicable diseases/high-risk pathogens category, many CAHs did not have a plan to process Category A hazardous substances, and over a quarter of the facilities reported lacking an airborne infection isolation room to isolate patients with a suspected or confirmed airborne pathogen (45%; 17 of 38 and 26%; 10 of 39, respectively).

## Discussion

The participants of this study helped identify overarching gaps in two categories. The first is related to audit and feedback practices. The intent of performing clinical audits is to provide a systematic review of healthcare staff performance based on evidence-based guidelines, adherence to recommendations, and may include measures of structures, processes, and/or outcomes of care.^22,23^ Feedback from the clinical audit process should provide a clear, constructive, non-punitive message that “directs the professionals’ attention to actionable, achievable tasks intended to improve patient care.”^22^

Challenges associated with conducting audits and feedback in CAHs may be associated with human resources availability and knowledge limitations, such as the lack of expertise in developing an audit and feedback program, training staff to perform audits and feedback, and identifying evidence-based guidelines supporting IPC practices and patient care interventions.^24^ Depending on the size of the CAH, the infection preventionist may be a “team of one” who is responsible for identifying reliable resources to develop specific audit and feedback tools.^25^ Access to evidence-based resources, guidance recommendations, and standards may also be a barrier for CAH infection preventionists if purchase-for-access or membership to a professional organization is required. New or inexperienced infection preventionists may not even be aware of existing free resources. Our previous work with 36 Nebraska CAHs during 2015–2017 identified that only 5.5% of CAH infection preventionists were board certified and 19.4% had no IPC training (unpublished data). It is essential for healthcare organizations to recognize the importance of developing a budget to support their program including professional development of IPC team.

The second overarching gap is competency-based training. Competency-based training is a framework for “designing and implementing education that focuses on the desired performance characteristics of health care professionals.”^26^ Specific performance characteristics identified during an educational program directly translate to outcomes or performance measures utilized in audit and feedback tools. The connection between competency-based training and audit and feedback practices assists in establishing the core elements necessary for a robust IPC program.

Infection preventionists working in CAHs may fulfill multiple roles within the organization, such as employee/occupational health manager, or quality/risk manager.^27^ Competing responsibilities may diminish the time that can be spent on essential elements of an IPC program including conducting audits and training. Additionally, support services such as secretarial, data, and electronic medical record support may be limited; thus, requiring the infection preventionist to dedicate additional time to the administrative functions of the IPC program.^28^

Majority of CAHs also lacked an efficient drug diversion program. Drug diversion poses significant challenges and has wide-ranging implications including putting patients at risk for healthcare-associated infections.^29^ Addressing drug diversion requires a multidimensional approach that involves collaboration among IPC professionals, pharmacy departments, human resources, and regulatory bodies.^30,31^ By identifying potential infection risks, implementing robust diversion prevention strategies, comprehensive education and training programs, and establishing robust monitoring systems, facilities can strive to ensure patient safety, healthcare provider safety, and maintain the integrity of the healthcare system.^30^ Due to their expertise in infection surveillance and outbreak response, IPC programs can play a vital role when drug diversion is suspected and therefore, should contribute to the development of drug diversion program for the facility.

To address IPC challenges in CAHs, we propose several key actions. First, the development and dissemination of tailored IPC guidelines considering CAHs unique limitations, such as limited resources and geographic isolation. Second, implementation of targeted education and training programs to raise awareness, enhance knowledge and skills, and foster a culture of patient safety among healthcare personnel. Third, promoting collaboration and networking among CAHs through regional or statewide communities of practice and focus groups for sharing best practices and innovative IPC strategies. Lastly, ensuring access to technical assistance and resources, including expert consultation, online toolkits, webinars, and support from state health departments, infection prevention organizations, and federal agencies. These actions aim to improve IPC practices in CAHs and enhance patient care.

Small sample size and lack of demographic data from respondents are the major limitations of the study. The very low response rate from two out of four states severely limits the generalizability of study findings. The reasons for variations among state response rates are likely multifactorial. The team that developed and disseminated the survey has longstanding relationships with local public health departments and hospitals across Nebraska and was able to leverage those relationships to encourage participation in this survey. These direct recruitment efforts contributed to a higher response rate in the state of Nebraska. Secondly, we requested public health departments and other stakeholders in each state to assist us in disseminating the surveys and getting the word out. However, it was up to the various partners to choose their own strategy for making the CAHs aware of the survey and encouraging them to participate. Finally, since there were variations in statewide COVID-19 responses in different states,^32^ COVID-19 burden or resource constraints of CAH at the time of the survey in various states may also be different, making them either more or less likely to respond to the survey.^25,33,34^ The timing of the needs assessment survey could also have had significant implications on the study’s outcomes as it was assessing perceived needs of participants during COVID-19 pandemic and the participants may already have worked on improving various aspects of their IPC programs.^35^ It is interesting that even though the survey was conducted during a pandemic, the response rate for questions related to high-risk pathogens and serious communicable diseases was lower than for questions from other categories. This could be attributed to several factors including limited exposure/experience, lack of prioritization of efforts in this area due to resource constraints, and/or low level of perceived relevance for their setting.^36,37^ Hence, this study may have underestimated IPC practice gaps in the category of high-risk pathogens and serious communicable diseases and there may be more need for resources in this area.

In summary, this study provided insight into IPC program areas requiring focused attention and resources needed by infection preventionists in CAHs to help improve IPC practices and prioritize resource development and dissemination. Future studies with larger sample size and collection of additional demographic information (e.g. hours spent by infection preventionist on IPC activities, years of experience, facility size, affiliation, and certification) to identify the needs of CAH infection preventionists with various levels of expertise and experience are needed. Studies specifically focused on assessing the needs of CAHs related to safely performing initial assessment and care for patients under investigation for high-risk pathogens and serious communicable diseases will also be helpful. It would also be valuable to explore the relationship between self-assessed gaps and external assessments, such as the Joint Commission surveys, to gain a more comprehensive understanding of IPC practices and their alignment with regulatory standards and guidelines.

## Supporting information

Soma et al. supplementary material Soma et al. supplementary material

## Supplementary material

For supplementary material accompanying this paper visit http://doi.org/10.1017/ash.2024.32. click here to view supplementary material
