# Supplementary material for: Resources needed by critical access hospitals to address identified infection prevention and control program gaps
Source: Antimicrob Steward Healthc Epidemiol. 2024 Mar 15;4(1):e34. doi: 10.1017/ash.2024.32 (PMC10945943; doi:10.1017/ash.2024.32)
Supplement: Soma et al. supplementary material [file S2732494X24000329sup001.pdf]

National Infection Control Strengthening Project Self-Assessment for Small and Rural Hospitals

Please take a few minutes to complete the important survey below.

Please note that the survey includes three different sections.

You may bookmark this page to return to the survey, OR you can have the survey link emailed to you by providing your email address (prompted when you hit the "Save and Return Later" button). If you do not receive an email from rito@unmc.edu soon afterward, please check your Junk Email folder.

If you have any technical difficulties, please reach out to Mounica Soma at msoma@nebraskamed.com

Thank you!

Facility Name

____

Please enter primary contact information of an individual completing the survey

---

Name

____

Email

____

Contact Number

____

Section I
The following questions are related to the infection control program and infrastructure at your facility.

---

1) Does your facility have respiratory etiquette stations and signage posted in visible locations for patients/visitors to self-identify and isolate? (e.g. "If you have a fever and cough or rash, please wear this mask.")

◯ Yes ◯ No ◯ Not Sure

If yes, what additional resources does your facility need to improve respiratory etiquette signage for patients/visitors to self-identify and isolate? (Select all that apply)

☐ Funding to stock PPE and signage stations
☐ Reliable supply chain for PPE
☐ Regulatory directive
☐ Administrative buy-in
☐ Nationally driven/standardized signage
☐ Other

If Other, please explain

____

If no, what resources does your facility need to be able to develop respiratory etiquette signage for patients/visitors to self-identify and isolate? (Select all that apply)

☐ Funding to stock PPE/signage stations
☐ Reliable supply chain for PPE
☐ Regulatory directive
☐ Administrative buy-in
☐ Nationally driven/standardized signage
☐ Other

If Other, please explain

____

2) Does your facility have hand hygiene stations and signage posted in visible locations for patients/visitors to utilize (e.g., alcohol-rub (ABHR))?

○ Yes ○ No ○ Not Sure

If yes, what additional resources does your facility need to improve the availability of ABHR for patient/visitor use? (Select all that apply)

☐ Funding to stock ABHR/cover cost of ABHR
☐ Reliable supply chain for ABHR
☐ Regulatory directive
☐ Administrative buy-in
☐ Nationally driven/standardized signage
☐ Other

If Other, please explain

____

If no, what resources does your facility need to improve the availability of ABHR for patient/visitor use? (Select all that apply)

☐ Funding to stock ABHR/cover cost of ABHR
☐ Reliable supply chain for ABHR
☐ Regulatory directive
☐ Administrative buy-in
☐ Nationally driven standard signage
☐ Other

If other, please explain

____

3) Are supplies necessary for adherence to PPE recommendations available and located near point the of use in your facility?

○ Yes ○ No ○ Not Sure

If yes, what additional resources do you need to improve PPE supply and availability at or near the point of use? (Select all that apply)

- ☐ A checklist of what PPE supplies are needed in each location
- ☐ Storage ideas/options for storing PPE near point of use
- ☐ Signage to identify where PPE is stored for clinician recognition and adherence
- ☐ Other
- ☐ PPE supplies are stored at point of use, we need no resources to improve

If Other, please explain

____

If no, what resources do you need to improve PPE supply and availability at or near the point of use? (Select all that apply)

- ☐ A checklist of what PPE supplies are needed in each location
- ☐ Storage ideas/options for storing PPE near point of use
- ☐ Signage to identify where PPE is stored for clinician recognition and adherence
- ☐ Other

If Other, please explain

____

4) When new equipment or products will be purchased or introduced at your facility, is your facility IP program consulted to ensure appropriate use, implementation, and development/incorporation into policies and procedures?

◯ Yes ◯ No ◯ Not Sure

If yes, what additional resources will you need to improve the incorporation of IP program consultation as a part of new purchase or product implementation? (Select all that apply)

- ☐ A procedure template for new products/purchases that incorporates IP program consultation
- ☐ Leadership buy-in
- ☐ Focus of regulatory authorities during surveys (e.g., staff education, appropriate cleaning/disinfection/sterilization)
- ☐ Other
- ☐ Our IP is consulted every time, no improvements needed to our program

If Other, please explain

____

If no, what resources will you need to improve the incorporation of IP program consultation as a part of new purchase or product implementation? (Select all that apply)

- ☐ A procedure template for new products/purchases that incorporates IP program consultation
- ☐ Leadership buy-in
- ☐ Focus of regulatory authorities during surveys (e.g., staff education, appropriate cleaning/disinfection/sterilization)
- ☐ Other

If Other, please explain

____

5) Does your hospital have a surveillance program to monitor incidence of epidemiologically important organisms (e.g., CRE) and targeted healthcare-associated infections (HAI)?

◯ Yes ◯ No ◯ Not Sure

If yes, what additional resources do you need to improve your surveillance program that monitors for incidents of epidemiologically important organisms and targeted HAIs? (Select all that apply)

☐ Lab system notification to IP when targeted organisms are identified
☐ Protected time for real time surveillance and data mining
☐ Basic infrastructure and/or office space (with computers, fax, internet access, supplies, and relevant software programs)
☐ Other
☐ Our surveillance program does not need improvement

If Other, please explain

____

If no, what resources do you need to be successful in developing a surveillance program that monitors for incident of epidemiologically important organisms and targeted HAIs? (Select all that apply)

☐ Lab system notification to IP when targeted organisms are identified
☐ Protected time for real time surveillance and data mining
☐ Basic infrastructure and/or office space (with computers, fax, internet access, supplies, and relevant software programs)
☐ Other

If Other, please explain

____

6) Does your hospital utilize surveillance data to implement corrective actions when transmission of epidemiologically important organisms or increased rates or persistently elevated rates of healthcare-associated infections are detected? (Corrective action example: Elevated BSI rate investigation uncovers a new device in use with central lines, without appropriate training for all staff. You pull the use of this new device until the further investigation and proper training on the device can occur.)

◯ Yes ◯ No ◯ Not Sure

If yes, what additional resources do you need to improve your surveillance program to ensure corrective action occurs when transmission or increased rate or persistently elevated rates of HAI are detected? (Select all that apply)

☐ Lab system notification to IP when targeted organisms are identified
☐ Data mining system in which notification alerts can be set for targeted organisms
☐ Protected IP time to do surveillance and corrective actions
☐ Access to local or state level expert to discuss strategies
☐ Leadership involvement in decision making for corrective actions
☐ Other
☐ Our surveillance program incorporates corrective action, no improvement needed

If Other, please explain

____

If no, what resources do you need to be successful in using surveillance data to implement corrective actions when transmission or increased rate or persistently elevated rates of HAI are detected? (Select all that apply)

☐ Lab system notification to IP when targeted organisms are identified
☐ Data mining system in which notification alerts can be set for targeted organisms
☐ Protected IP time to do surveillance and corrective actions
☐ Access to local or state level expert to discuss strategies
☐ Leadership involvement in decision making for corrective actions
☐ Other

If Other, please explain

____

7) Does your hospital implement infection control measures relevant to construction, renovation, demolition, and repairs including performance of an infection control risk assessment (ICRA) before a project gets underway?

○ Yes ○ No ○ Not Sure

If yes, what additional resources do you need to improve implementation of IC measures relevant to construction, renovation, demolition, and repairs, and performance of an ICRA prior to project initiation? (Select all that apply)

☐ A procedure template that outlines the process, including the ICRA template and a timeline for what steps should happen when in the process
☐ Administrative buy-in for performance of an ICRA with each project
☐ Focus of regulatory authorities during surveys related to construction/renovation/demolition/repair projects
☐ Access to ICRA training courses for IPs
☐ Access to ICRA training courses for contractors
☐ Other
☐ We have a well-developed and implanted ICRA program, no improvements are needed

If Other, please explain

____

If no, what resources do you need to be successful in implementing IC measures relevant to construction, renovation, demolition, and repairs, and performance of an ICRA prior to project implementation? (Select all that apply)

☐ A procedure template that outlines the process, including the ICRA template and a timeline for what steps should happen when in the process
☐ Administrative buy-in for performance of an ICRA with each project
☐ Focus of regulatory authorities during surveys related to construction/renovation/demolition/repair projects
☐ Access to ICRA training courses for IPs
☐ Access to ICRA training courses for contractors
☐ Other

If Other, please explain

____

8) Does your facility have a water management plan/program that includes IP consultation?

○ Yes ○ No ○ Not Sure

If yes, what additional resources do you need to improve your facility's water management plan/program that includes consultation with IP? (Select all that apply)

☐ Administrative support
☐ Financial support for FTE to create and execute the water management plan
☐ Access to guidance on assessing/evaluating a water management plan/program
☐ Training for personnel in all aspects of water management plan/program
☐ Other
☐ Our water management program is well-developed and implemented, not improvements are needed

If Other, please explain

____

If no, what resources do you need to develop a water management plan/program at your facility? (Select all that apply)

☐ Administrative support
☐ Financial support for FTE to develop and execute the water management plan
☐ Access to guidance on creating a water management plan
☐ Training for personnel in aspects of water management
☐ Other

If Other, please explain

____

9) Does your facility have a respiratory protection program that that emphasizes preventing the transmission of aerosol transmissible diseases to personnel?

◯ Yes ◯ No ◯ Not Sure

If yes, what additional resources do you need to improve your respiratory protection program? (Select all that apply)

☐ Financial support for FTE to develop/sustain the program
☐ Administrative support and approval to implement a fit-test program or contract a third party for fit-testing
☐ Access to guidance on developing a respiratory protection program
☐ Other
☐ Our facility respiratory protection program is well-developed and implemented, not improvements are needed

If Other, please explain

____

If no, what resources does your facility need to be able to develop a respiratory protection program? (Select all that apply)

☐ Financial support for FTE to develop and sustain the program
☐ Administrative support and approval to implement a fit-test program or contract a third party for fit-testing
☐ Access to guidance on developing a respiratory protection program
☐ Other

If Other, please explain

____

10) Does your facility perform an Infection Prevention and Control Program annual infection risk assessment that evaluates and prioritizes potential risks for infections, contamination, & exposures and the program's preparedness to eliminate or mitigate risks?

◯ Yes ◯ No ◯ Not Sure

If yes, what additional resources do you need to improve your annual infection risk assessment? (Select all that apply)

- ☐ Analysis/results of assessment data of our current operations (e.g. hand hygiene audit data, infection surveillance data, etc.)
- ☐ Educational resources to train personnel in performing an infection risk assessment
- ☐ A standard template for performing an infection risk assessment with built-in formula's for scoring each risk
- ☐ A guide to know what risk topics should be included in the risk assessment
- ☐ Leadership support for performing an infection risk assessment
- ☐ An interdisciplinary team to contribute to performance of the assessment
- ☐ Dedicated FTE for completing the infection risk assessment
- ☐ Other
- ☐ Our annual risk assessment is well-developed and implemented, not improvements are needed

If Other, please explain

____

If no, what resources do you need to develop an annual infection risk assessment? (Select all that apply)

- ☐ Analysis/results of assessment data of our current operations (e.g. hand hygiene audit data, infection surveillance data, etc.)
- ☐ Educational resources to train personnel in performing an infection risk assessment
- ☐ A standard template for performing an infection risk assessment with built-in formula's for scoring each risk
- ☐ A guide to know what risk topics should be included in the risk assessment
- ☐ Leadership support for performing an infection risk assessment
- ☐ An interdisciplinary team to contribute to performance of the assessment
- ☐ Dedicated FTE for completing the infection risk assessment
- ☐ Other

If Other, please explain

____

11) Does your facility have a drug diversion program that includes consultation with the IP (when drug tampering is suspected or identified) to assess patient safety risks?

◯ Yes ◯ No ◯ Not Sure

If yes, what additional resources do you need to improve your drug diversion program at your facility? (Select all that apply)

- ☐ A guide for creating and implementing a drug diversion program
- ☐ An educational resource to train personnel on drug diversion
- ☐ A policy/protocol template inclusive of steps to follow in an investigation of drug tampering
- ☐ Leadership support for creation of a drug diversion program
- ☐ Directive from an accrediting/regulatory group (e.g. CMS)
- ☐ Other
- ☐ Our facility's drug diversion program is well-developed and implemented, no improvements are needed

If Other, please explain

____

If no, what resources do you need to develop a drug diversion program at your facility?(Select all that apply)

- ☐ A guide for creating and implementing a drug diversion program
- ☐ An educational resource to train personnel on drug diversion
- ☐ A policy/protocol template inclusive of steps to follow in an investigation of drug tampering
- ☐ Leadership support for creation of a drug diversion program
- ☐ Directive from an accrediting/regulatory group (e.g. CMS)
- ☐ Other

If Other, please explain

____

12) Does your facility have procedures outlining hospital response in the event of a reprocessing error or failure (e.g., sterilization and/or high-level disinfection)?

○ Yes ○ No ○ Not Sure

If yes, what additional resources does your facility need to improve its response plan in the event of a reprocessing error or failure? (Select all that apply)

- ☐ Resources to train additional personnel to identify and report reprocessing errors/failures
- ☐ Educational resources to train additional personnel on how to perform reprocessing error/failure investigations
- ☐ Standardized tool/template to document investigation process and findings
- ☐ A tool or database for storing investigation findings
- ☐ Dedicated FTE for educating and training staff who reprocess medical devices
- ☐ Leadership support
- ☐ Other
- ☐ We have a well-designed and implemented response plan, no improvements are needed

If Other, please explain

____

If no, what resources does your facility need to develop its response plan in the event of a reprocessing error or failure? (Select all that apply)

- ☐ Resources to train additional personnel to identify and report reprocessing errors/failures
- ☐ Educational resources to train additional personnel on how to perform reprocessing error/failure investigations
- ☐ Standardized tool/template to document investigation process and findings
- ☐ A tool or database for storing investigation findings
- ☐ Dedicated FTE for educating and training staff who reprocess medical devices
- ☐ Leadership support
- ☐ Other

If Other, please explain

____

13) Does your facility have procedures outlining hospital response in the event of a product recall? NOTE: Product recalls can include patient care equipment, pharmaceuticals, or FDA medical devices.

◯ Yes ◯ No ◯ Not Sure

If yes, what additional resources does your facility need to improve its response in the event of a product recall? (Select all that apply)

- ☐ Centralized department to receive recall notices (via mail, email, or online database/recall system)
- ☐ Resources to train additional personnel on how to determine if recalled product has been purchased and/or used in the facility
- ☐ Standardized tool/template to document recall activities (e.g., pulled from inventory and returned to manufacturer)
- ☐ A tool or database for storing product recall activities and information
- ☐ Dedicated FTE to monitor for product recalls
- ☐ Leadership support
- ☐ Other
- ☐ We have a well-designed and implemented product recall plan, no improvements are needed

If Other, please explain

____

If no, what resources does your facility need to develop its response in the event of a product recall? (Select all that apply)

- ☐ Centralized department to receive recall notices (via mail, email, or online database/recall system)
- ☐ Resources to train additional personnel on how to determine if recalled product has been purchased and/or used in the facility
- ☐ Standardized tool/template to document recall activities (e.g., pulled from inventory and returned to manufacturer)
- ☐ A tool or database for storing product recall activities and information
- ☐ Dedicated FTE to monitor for product recalls
- ☐ Leadership support
- ☐ Other

If Other, please explain

____

Section IIA--Competency-based training
Competency-based training definition:
A course of instruction that provides both information and skills practice, which is based upon clearly stated and measurable instructional objectives, and which requires demonstration (competence) of the achievement of a particular standard of skills and knowledge for satisfactory completion.

1) Does your facility provide a competency-based training program related to hand hygiene?

◯ Yes ◯ No ◯ Not Sure

If yes, what additional resources do you need to be successful in strengthening your competency-based training program for hand hygiene (to include hand washing and alcohol-based hand rub (ABHR))? (choose all that apply)

☐ Educational resources for training additional personnel (e.g., webinar, videos, slide sets, etc.)
☐ Educational and training supplies and equipment
☐ Post-educational assessment tools to verify knowledge, skills, and competence
☐ On-site or on-staff personnel with adequate subject matter expertise
☐ Dedicated FTE for a formal educator
☐ Time allotted during employee orientation/onboarding for this topic
☐ Annual education, competency verification, and documentation devoted to this topic
☐ Directive from accrediting/regulatory group (e.g., CMS)
☐ Other
☐ We have a well-designed and implemented program, no improvements are needed

If other, please explain

____

If no, what resources do you need to develop and provide a competency-based training program for hand hygiene (to include alcohol-based hand rub and soap and water)? (Choose all that apply)

☐ Educational resources for training personnel (e.g., webinar, videos, slide sets, etc.)
☐ Educational and training supplies and equipment
☐ Post-educational assessment tools to verify knowledge, skills, and competence
☐ On-site or on-staff personnel with adequate subject matter expertise
☐ Dedicated FTE for a formal educator
☐ Time allotted during employee orientation/onboarding for this topic
☐ Annual education, competency verification, and documentation devoted to this topic
☐ Directive from accrediting/regulatory group (e.g., CMS)
☐ Other

If other, please explain

____

2) Does you facility provide a competency-based training program for all personnel who require knowledge on proper PPE selection, based on pathogen transmission? In other words, training them on how to determine what PPE would be appropriate to wear based on a patient's infection diagnosis and pathogen presence.

◯ Yes ◯ No ◯ Not Sure

If yes, what additional resources do you need to strengthen your competency-based training program for proper PPE selection? (Choose all that apply)

☐ Educational resources for training additional personnel (e.g., webinar, videos, slide sets, etc.)
☐ Educational and training supplies and equipment
☐ Post-educational assessment tools to verify knowledge, skills, and competence
☐ On-site or on-staff personnel with adequate subject matter expertise
☐ Dedicated FTE for a formal educator
☐ Time allotted during employee orientation/onboarding for this topic
☐ Annual education, competency verification, and documentation devoted to this topic
☐ Directive from accrediting/regulatory group (e.g., CMS)
☐ Other
☐ We have a well-designed and implemented program, no improvements are needed

If other, please explain

____

If no, what resources do you need to develop and provide a competency-based training program for proper PPE selection? Choose all that apply)

- ☐ Educational resources for training personnel (e.g., webinar, videos, slide sets, etc.)
- ☐ Educational and training supplies and equipment
- ☐ Post-educational assessment tools to verify knowledge, skills, and competence
- ☐ On-site or on-staff personnel with adequate subject matter expertise
- ☐ Dedicated FTE for a formal educator
- ☐ Time allotted during employee orientation/onboarding for this topic
- ☐ Annual staff education, competency verification, and documentation devoted to this topic
- ☐ Directive from accrediting/regulatory group (e.g., CMS)
- ☐ Other

If other, please explain

____

3) Does your facility provide a competency-based training program for all personnel who require knowledge on proper donning and doffing of PPE? NOTE: PPE includes gown/gloves/eye or face protection/masks/respirators (hair and/or shoe coves, if applicable).

○ Yes ○ No ○ Not Sure

If yes, what additional resources do you need to strengthen your competency-based training program for proper donning and doffing of PPE? (Select all that apply)

- ☐ Educational resources for training additional personnel (e.g., webinar, videos, slide sets, etc.)
- ☐ Educational and training supplies and equipment
- ☐ Post-educational assessment tools to verify knowledge, skills, and competence
- ☐ On-site or on-staff personnel with adequate subject matter expertise
- ☐ Dedicated FTE for a formal educator
- ☐ Time allotted during employee orientation/onboarding for this topic
- ☐ Annual education, competency verification, and documentation devoted to this topic
- ☐ Directive from accrediting/regulatory group (e.g., CMS)
- ☐ Other
- ☐ We have a well-designed and implemented program, no improvements are needed

If Other, please explain

____

If no, what resources do you need to develop and provide a competency-based training program for proper donning and doffing of PPE? (Select all that apply)

- ☐ Educational resources for training personnel (e.g., webinar, videos, slide sets, etc.)
- ☐ Educational and training supplies and equipment
- ☐ Post-educational assessment tools to verify knowledge, skills, and competence
- ☐ On-site or on-staff personnel with adequate subject matter expertise
- ☐ Dedicated FTE for a formal educator
- ☐ Time allotted during employee orientation/onboarding for this topic
- ☐ Annual education, competency verification, and documentation devoted to this topic
- ☐ Directive from accrediting/regulatory group (e.g., CMS)
- ☐ Other

If Other, please explain

____

4) Does your facility provide a competency-based training program for safe injection practices? NOTE: Practices include preparation and/or administration of injections and parenteral infusions.

◯ Yes ◯ No ◯ Not Sure

If yes, what additional resources do you need to strengthen your competency-based training program for safe injection practices? (Select all that apply)

- ☐ Educational resources for training additional personnel (e.g., webinar, videos, slide sets, etc.)
- ☐ Educational and training supplies and equipment
- ☐ Post-educational assessment tools to verify knowledge, skills, and competence
- ☐ On-site or on-staff personnel with adequate subject matter expertise
- ☐ Dedicated FTE for a formal educator
- ☐ Time allotted during employee orientation/onboarding for this topic
- ☐ Annual education, competency verification, and documentation devoted to this topic
- ☐ Directive from accrediting/regulatory group (e.g., CMS)
- ☐ Other
- ☐ We have a well-designed and implemented program, no improvements are needed

If Other, please explain

____

If no, what resources do you need to develop and provide a competency-based training program for safe injection practices? (Select all that apply)

- ☐ Educational resources for training personnel (e.g., webinar, videos, slide sets, etc.)
- ☐ Educational and training supplies and equipment
- ☐ Post-educational assessment tools to verify knowledge, skills, and competency
- ☐ On-site or on-staff personnel with adequate subject matter expertise
- ☐ Dedicated FTE for a formal educator
- ☐ Time allotted during employee orientation/onboarding for this topic
- ☐ Annual education, competency verification, and documentation devoted to this topic
- ☐ Directive from accrediting/regulatory group (e.g., CMS)
- ☐ Other

If Other, please explain

____

5) Does your facility provide a competency-based training program for personnel who insert and maintain (care for) (indwelling) urinary catheters?

◯ Yes ◯ No ◯ Not Sure

If yes, what additional resources do you need to strengthen your competency-based training program for insertion and maintenance of urinary catheters? (Select all that apply)

- ☐ Educational resources for training additional personnel (e.g., webinar, videos, slide sets, etc.)
- ☐ Educational and training supplies and equipment (e.g., anatomical models)
- ☐ Post-educational assessment tools to verify knowledge, skills, and competency
- ☐ On-site or on-staff personnel with adequate subject matter expertise
- ☐ Dedicated FTE for a formal educator
- ☐ Time allotted during employee orientation/onboarding for this topic
- ☐ Annual education, competency verification, and documentation devoted to this topic
- ☐ Directive from accrediting/regulatory group (e.g., CMS)
- ☐ Other
- ☐ We have a well-designed and implemented program, no improvements are needed

If Other, please explain

____

If no, what resources do you need to develop and provide a competency-based training program for personnel who insert and maintain urinary catheters? (Select all that apply)

- ☐ Educational resources for training personnel (e.g., webinar, videos, slide sets, etc.)
- ☐ Educational and training supplies and equipment (e.g., anatomical models)
- ☐ Post-educational assessment tools to verify knowledge, skills, and competency
- ☐ On-site or on-staff personnel with adequate subject matter expertise
- ☐ Dedicated FTE for a formal educator
- ☐ Time allotted during employee orientation/onboarding for this topic
- ☐ Annual education, competency verification, and documentation devoted to this topic
- ☐ Directive from accrediting/regulatory group (e.g., CMS)
- ☐ Other

If Other, please explain

____

6) Does your facility provide a competency-based training program for personnel who insert and maintain (care for) central venous catheters (CVCs)?

○ Yes ○ No ○ Not Sure

If yes, what additional resources do you need to strengthen your competency-based training program for CVC insertion and maintenance? (Select all that apply)

- ☐ Educational resource to train additional personnel (e.g. webinar, videos, slide sets, etc.)
- ☐ Educational and training supplies and equipment
- ☐ Post-educational assessment tools to verify knowledge, skills, and competence
- ☐ On-site or on-staff personnel with adequate subject matter expertise
- ☐ Dedicated FTE for a formal educator
- ☐ Time allotted during employee orientation/onboarding for this topic
- ☐ Annual education, competency verification, and documentation devoted to this topic
- ☐ Directive from accrediting/regulatory group (e.g., CMS)
- ☐ Other
- ☐ We have a well-designed and implemented program, no improvements are needed

If Other, please explain

____

If no, what resources do you need to develop and provide a competency-based training program for CVC insertion and maintenance? (Select all that apply)

☐ Educational resources for training personnel (e.g., webinar, videos, slide sets, etc.)
☐ Educational and training supplies and equipment
☐ Post-educational assessment tools to verify knowledge, skills, and competence
☐ On-site or on-staff personnel with adequate subject matter expertise
☐ Dedicated FTE for a formal educator
☐ Time allotted during employee orientation/onboarding for this topic
☐ Annual education, competency verification, and documentation devoted to this topic
☐ Directive from accrediting/regulatory group (e.g., CMS)
☐ Other

If Other, please explain

____

7) Does your facility provide a competency-based training program for all personnel who perform environmental cleaning? NOTE: Environmental cleaning includes non-critical patient care equipment, mobile devices, and other electronics (e.g., point-of-care devices, mobile work stations).

◯ Yes ◯ No ◯ Not Sure

If yes, what additional resources do you need to strengthen your competency-based training program for environmental cleaning? (Select all that apply)

☐ Educational resources for training additional personnel (e.g., webinar, videos, slide sets, etc.)
☐ Educational and training supplies and equipment
☐ Post-educational assessment tools to verify knowledge, skills, and competence
☐ On-site or on-staff personnel with adequate subject matter expertise
☐ Dedicated FTE for a formal educator
☐ Time allotted during employee orientation/onboarding for this topic
☐ Annual education, competency verification, and documentation devoted to this topic
☐ Directive from accrediting/regulatory group (e.g., CMS)
☐ Other
☐ We have a well-designed and implemented program, no improvements are needed

If Other, please explain

____

If no, what resources do you need to develop and provide a competency-based training program for environmental cleaning?

☐ Educational resources for training personnel (e.g., webinar, videos, slide sets, etc.)
☐ Educational and training supplies and equipment
☐ Post-educational assessment tools to verify knowledge, skills, and competence
☐ On-site or on-staff personnel with adequate subject matter expertise
☐ Dedicated FTE for a formal educator
☐ Time allotted during employee orientation/onboarding for this topic
☐ Annual education, competency verification, and documentation devoted to this topic
☐ Directive from accrediting/regulatory group (e.g., CMS)
☐ Other

If Other, please explain

____

8) Does your facility provide a competency-based training program for personnel who reprocess semi-critical devices, that is, perform high-level disinfection (HLD)? NOTE: Examples of devices you might perform HLD for include: endoscopes, speculums, ultrasound probes, or devices that can't undergo heat sterilization.

◯ Yes ◯ No ◯ Not Sure

If yes, what additional resources do you need to strengthen your competency-based training program for reprocessing semi-critical devices? (Select all that apply)

- ☐ Educational resources for training additional personnel (e.g., webinar, videos, slide sets, etc.)
- ☐ Educational and training supplies and equipment
- ☐ Post-educational assessment tools to verify knowledge, skills, and competence
- ☐ On-site or on-staff personnel with adequate subject matter expertise
- ☐ Dedicated FTE for a formal educator
- ☐ Time allotted during employee orientation/onboarding for this topic
- ☐ Annual education, competency verification, and documentation devoted to this topic
- ☐ Directive from accrediting/regulatory group (e.g., CMS)
- ☐ Other
- ☐ We have a well-designed and implemented program, no improvements are needed

If Other, please explain

____

If no, what resources do you need to develop and provide a competency-based training program for reprocessing semi-critical devices? (Select all that apply)

- ☐ Educational resources for training personnel (e.g., webinar, videos, slide sets, etc.)
- ☐ Educational supplies and equipment
- ☐ Post-educational assessment tool to verify knowledge, skills, and competence
- ☐ On-site or on-staff personnel with adequate subject matter expertise
- ☐ Dedicated FTE for a formal educator
- ☐ Time allotted during employee orientation/onboarding for this topic
- ☐ Annual education, competency verification, and documentation devoted to this topic
- ☐ Directive from accrediting/regulatory group (e.g., CMS)
- ☐ Other
- ☐ Do not reprocess any semi-critical devices here (do not perform HLD)

If Other, please explain

____

9) Does your facility provide a competency-based training program for personnel who reprocess critical devices - that is, perform sterilization (e.g., surgical instruments)?

◯ Yes ◯ No ◯ Not Sure

If yes, what additional resources do you need to strengthen your competency-based training program for reprocessing critical devices? (Select all that apply)

- ☐ Educational resources for training additional personnel (e.g., webinar, videos, slide sets, etc.)
- ☐ Educational and training supplies and equipment
- ☐ Post-educational assessment tools to verify knowledge, skills, and competence
- ☐ On-site or on-staff personnel with adequate subject matter expertise
- ☐ Dedicated FTE for a formal educator
- ☐ Time allotted during employee orientation/onboarding for this topic
- ☐ Annual education, competency verification, and documentation devoted to this topic
- ☐ Directive from accrediting/regulatory group (e.g., CMS)
- ☐ Other
- ☐ We have a well-designed and implemented program, no improvements are needed

If Other, please explain

____

If no, what resources do you need to develop and provide a competency-based training program for reprocessing critical devices? (Select all that apply)

- ☐ Educational resource to train personnel (e.g., webinar, videos, slide sets, etc.).
- ☐ Educational supplies and equipment
- ☐ Post-educational assessment tools to verify knowledge, skills, and competence
- ☐ Directive from accrediting/regulatory group (e.g., CMS)
- ☐ On-site or on-staff personnel with adequate subject matter expertise
- ☐ Dedicated FTE for a formal educator
- ☐ Time allotted during employee orientation/onboarding for this topic
- ☐ Annual education, competency verification, and documentation devoted to this topic
- ☐ Other
- ☐ We do not reprocess any critical devices (do not perform sterilization)

If Other, please explain

____

10) Does your facility provide a competency-based training program for adherence to recommended infection control (IC) practices for surgical site infection (SSI) prevention? NOTE: IP practices include, but are not limited to preoperative surgical scrub/hand hygiene, appropriate use of surgical drapes, appropriate PPE selection and use, adherence to aseptic technique, and maintaining a sterile field.

◯ Yes ◯ No ◯ Not Sure

If yes, what additional resources do you need to strengthen your competency-based training program for IP practices related to surgical site infection (SSI) prevention? (Select all that apply)

- ☐ Educational resources for training additional personnel (e.g., webinar, videos, slide sets, etc.)
- ☐ Educational supplies and equipment
- ☐ Post-educational assessment tools to verify knowledge, skills, and competence
- ☐ On-site or on-staff personnel with adequate subject matter expertise
- ☐ Dedicated FTE for a formal educator
- ☐ Time allotted during employee orientation/onboarding for this topic
- ☐ Annual education, competency verification, and documentation devoted to this topic
- ☐ Directive from accrediting/regulatory group (e.g., CMS)
- ☐ Other
- ☐ We have a well-designed and implemented program, no improvements are needed

If Other, please explain

____

If no, what resources do you need to develop and provide a competency-based training program for IP practices related to surgical site infection (SSI) prevention? (Select all that apply)

☐ Educational resource to train personnel (e.g., webinar, videos, slide sets, etc.).
☐ Educational and training supplies and equipment
☐ Post-educational assessment tools to verify knowledge, skills, and competence
☐ Directive from accrediting/regulatory group (e.g., CMS)
☐ On-site or on-staff personnel with adequate subject matter expertise
☐ Dedicated FTE for a formal educator
☐ Time allotted during employee orientation/onboarding for this topic
☐ Annual education, competency validation, and documentation devoted to this topic
☐ Other
☐ We do not perform surgical procedures (do not monitor for SSIs)

If Other, please explain

____

11) Does your facility provide a competency-based training program for adherence to recommended infection control (IC) practices for C. difficile infection (CDI) prevention?

◯ Yes ◯ No ◯ Not Sure

If yes, what additional resources do you need to strengthen your competency-based training program for IP practices related to C. difficile infection (CDI) prevention? (Select all that apply)

☐ Educational resources for training additional personnel (e.g., webinar, videos, slide sets, etc.)
☐ Educational supplies and equipment
☐ Post-educational assessment tools to verify knowledge, skills, and competence
☐ On-site or on-staff personnel with adequate subject matter expertise
☐ Dedicated FTE for a formal educator
☐ Time allotted during employee orientation/onboarding for this topic
☐ Annual education, competency verification, and documentation devoted to this topic
☐ Directive from accrediting/regulatory group (e.g., CMS)
☐ Other
☐ We have a well-designed and implemented program, no improvements are needed

If Other, please explain

____

If no, what resources do you need to develop and provide a competency-based training program for IP practices related to C. difficile infection (CDI) prevention? (Select all that apply)

☐ Educational resource to train personnel (e.g., webinar, videos, slide sets, etc.).
☐ Educational and training supplies and equipment
☐ Post-educational assessment tools to verify knowledge, skills, and competence
☐ Directive from accrediting/regulatory group (e.g., CMS)
☐ On-site or on-staff personnel with adequate subject matter expertise
☐ Dedicated FTE for a formal educator
☐ Time allotted during employee orientation/onboarding for this topic
☐ Annual education, competency validation, and documentation devoted to this topic
☐ Other

If Other, please explain

____

Section IIB-- Audit and Feedback
Audit and Feedback Definition:
Audit: Measurement of work practice or performance, usually by observation, that is compared to professional standards (use of a checklist to measure compliance with expected performance).
Feedback: Sharing the results of the audit with the observed ndividual, in order to improve performance
(In order to answer "Yes", the respondent should have documentation of audits, results and feedback).

---

12) Does your facility perform audits and feedback on compliance with hand hygiene protocols/best practices (hand washing and alcohol-based hand rub (ABHR) use)?

◯ My facility performs both audit and feedback on hand hygiene
◯ My facility performs only audits of hand hygiene
◯ My facility does not perform audit or feedback of hand hygiene
◯ Not Sure

If yes, what additional recources do you need to strengthen your audit and feedback program for hand hygiene? (Select all that apply)

☐ Educational resources to train additional personnel to perform audits
☐ Educational resources to train additional personnel on how to provide and receive feedback
☐ Standardized hand hygiene audit tool (template or mobile app to assist audits)
☐ A tool or database for storing audit and feedback data
☐ Directive from accrediting/regulatory group (e.g. CMS)
☐ Dedicated FTE for performing audits and feedback
☐ Leadership support/expectation for audits and feedback to occur
☐ Other
☐ We have a well-designed and implemented program, no improvements are needed

---

If other, please explain

____

If no, what resources do you need to develop an audit and feedback program for hand hygiene? (Select all that apply)

☐ Educational resources to train personnel to perform audits
☐ Educational resources to train personnel on how to provide and receive feedback
☐ Standardized hand hygiene audit tool (template or mobile app to assist audits)
☐ A tool or database for storing audit and feedback data
☐ Directive from accrediting/regulatory group (e.g. CMS)
☐ Dedicated FTE for performing audits and feedback
☐ Leadership support/expectation for audits and feedback to occur
☐ Other

If other, please explain

____

---

13) Does your facility perform audits and feedback for adherence to proper selection of personal protective equipment (PPE)? NOTE: Selection of appropriate PPE based on patient's infection diagnosis and pathogen presence.

◯ My facility performs both audits and feedback on PPE selection
◯ My facility performs only audits of PPE selection
◯ My facility does not perform audit or feedback of proper PPE selection
◯ Not Sure

If yes, what additional resources do you need to improve/strengthen your audit and feedback program for PPE selection? (Select all that apply)

- ☐ Educational resources to train additional personnel to perform audits
- ☐ Educational resources to train additional personnel on how to provide and receive feedback
- ☐ Standardized PPE selection audit tool (template or mobile app to assist audits)
- ☐ A tool or database for storing audit and feedback data
- ☐ Directive from accrediting/regulatory group (e.g. CMS)
- ☐ Dedicated FTE for performing audits and feedback
- ☐ Leadership support/expectation for audits and feedback to occur
- ☐ Other
- ☐ We have a well-designed and implemented program, no improvements are needed

If other, please explain

____

If no, what resources do you need develop an audit and feedback program PPE selection? (Select all that apply)

- ☐ Educational resources to train personnel to perform audits
- ☐ Educational resources to train personnel on how to provide and receive feedback
- ☐ Standardized PPE selection audit tool (template or mobile app to assist audits)
- ☐ A tool or database for storing audit and feedback data
- ☐ Directive from accrediting/regulatory group (e.g. CMS)
- ☐ Dedicated FTE for performing audits and feedback
- ☐ Leadership support/expectation for audits and feedback to occur
- ☐ Other

If other, please explain

____

14) Does your facility perform audits and feedback for adherence to proper donning and doffing of PPE? NOTE: PPE includes gown/gloves/eye or face protection/masks/respirators (hair and/or shoe coves, if applicable).

- ◯ My facility performs both audits and feedback for proper donning and doffing of PPE
- ◯ My facility performs only audits for proper donning and doffing of PPE
- ◯ My facility does not perform audits or feedback for proper donning and doffing of PPE
- ◯ Not Sure

If yes, what additional resources do you need to strengthen your audit and feedback program for adherence to proper donning and doffing of PPE? (Select all that apply)

- ☐ Educational resources to train additional personnel to perform audits
- ☐ Educational resources to train additional personnel on how to provide and receive feedback
- ☐ Standardized PPE donning and doffing audit tool (template or mobile app to assist audits)
- ☐ A tool or database for storing audit and feedback data
- ☐ Directive from accrediting/regulatory group (e.g. CMS)
- ☐ Dedicated FTE for performing audits and feedback
- ☐ Leadership support/expectation for audits and feedback to occur
- ☐ Other
- ☐ We have a well-designed and implemented program, no improvements are needed

If other, please explain

____

If no, what resources do you need to develop an audit and feedback program for proper donning and doffing for PPE? (Select all that apply)

☐ Educational resources to train personnel to perform audits
☐ Educational resources to train personnel on how to provide and receive feedback
☐ Standardized PPE donning and doffing audit tool (template or mobile app to assist audits)
☐ A tool or database for storing audit and feedback data
☐ Directive from accrediting/regulatory group (e.g. CMS)
☐ Dedicated FTE for performing audits and feedback
☐ Leadership support/expectation for audits and feedback to occur
☐ Other

If other, please explain

____

15) Does your facility perform audits and feedback for safe injection practices? NOTE: Practices include preparation and/or administration of injections and parenteral infusions.

◯ My facility performs both audits and feedback for safe injection practices
◯ My facility only performs audits for safe injection practices
◯ My facility does not perform audits or feedback for safe injection practices
◯ Not Sure

If yes, what additional resources do you need to strengthen your audit and feedback program for adherence to safe injection practices? (Select all that apply)

☐ Educational resource to train additional personnel to perform audits
☐ Educational resource to train additional personnel on how to provide and feedback
☐ Standardized safe injection practices audit tool (template or mobile app to assist audits)
☐ A tool or database for storing audit and feedback data
☐ Directive from accrediting/regulatory group (e.g. CMS)
☐ Dedicated FTE for performing audits and feedback
☐ Leadership support/expectation for audits and feedback to occur
☐ Other
☐ We have a well-designed and implemented program, no improvements are needed

If Other, please explain

____

If no, what resources do you need to develop an audit and feedback program for safe injection practices? (Select all that apply)

☐ Educational resources to train personnel to perform audits
☐ Educational resources to train personnel on how to provide and receive feedback
☐ Standardized safe injection practices audit tool (template or mobile app to assist audits)
☐ A tool or database for storing audit and feedback data
☐ Directive from accrediting/regulatory group (e.g. CMS)
☐ Dedicated FTE for performing audits and feedback
☐ Leadership support/expectation for audits and feedback to occur
☐ Other

If Other, please explain

____

16) Does your facility perform audits and feedback for insertion and maintenance of (indwelling) urinary catheters?

◯ My facility performs both audits and feedback for insertion and maintenance of urinary catheters
◯ My facility only performs audits for insertion and maintenance of urinary catheters
◯ My facility does not perform audits or feedback for insertion and maintenance of urinary catheters
◯ Not Sure

If yes, what additional resources do you need to strengthen your audit and feedback program for insertion and maintenance of urinary catheters? (Select all that apply)

☐ Educational resource to train additional personnel to perform audits
☐ Educational resource to train additional personnel on how to provide and receive feedback
☐ Standardized urinary catheter insertion and maintenance audit tool (template or mobile app to assist audits)
☐ A tool or database for storing audit and feedback data
☐ Directive from accrediting/regulatory group (e.g. CMS)
☐ Dedicated FTE for performing audits and feedback
☐ Leadership support/expectation for audits and feedback to occur
☐ Other
☐ We have a well-designed and implemented program, no improvements are needed

If other, please explain

____

If no, what resources do you need to develop an audit and feedback program for insertion and maintenance of urinary catheters? (Select all that apply)

☐ Educational resources to train personnel to perform audits
☐ Educational resources to train personnel on how to provide and receive feedback
☐ Standardized urinary catheter insertion and maintenance audit tool (template or mobile app to assist audits)
☐ A tool or database for storing audit and feedback data
☐ Directive from accrediting/regulatory group (e.g. CMS)
☐ Dedicated FTE for performing audits and feedback
☐ Leadership support/expectation for audits and feedback to occur
☐ Other

If other, please explain

____

17) Does your facility perform audits and feedback for insertion and maintenance for central venous catheters (CVCs)?

◯ My facility performs both audits and feedback for insertion and maintenance for CVCs
◯ My facility only performs audits for insertion and maintenance for CVCs
◯ My facility does not perform audits or feedback of insertion and maintenance of CVCs
◯ Not Sure

If yes, what additional resources do you need to strengthen your audit and feedback program for insertion and maintenance of CVCs? (Select all that apply)

- ☐ Educational resources to train additional personnel to perform audits
- ☐ Educational resources to train additional personnel on how to provide and receive feedback
- ☐ Standardized insertion and maintenance audit tool for CVCs (template or mobile app to assist audits)
- ☐ A tool or database for storing audit and feedback data
- ☐ Directive from accrediting/regulatory group (e.g. CMS)
- ☐ Dedicated FTE for performing audits and feedback
- ☐ Leadership support/expectation for audits and feedback to occur
- ☐ Other
- ☐ We have a well-designed and implemented program, no improvements are needed

If other, please explain

____

If no, what resources do you need to develop an audit and feedback program for insertion and maintenance of CVCs? (Select all that apply)

- ☐ Educational resources to train personnel to perform audits
- ☐ Educational resources to train personnel on how to provide and receive feedback
- ☐ Standardized CVC insertion and maintenance audit tool (template or mobile app to assist audits)
- ☐ A tool or database for storing audit and feedback data
- ☐ Directive from accrediting/regulatory group (e.g. CMS)
- ☐ Dedicated FTE for performing audits and feedback
- ☐ Leadership support/expectation for audits and feedback to occur
- ☐ Other

If other, please explain

____

18) Does your facility perform audits and feedback on adherence to cleaning and disinfection procedures, including use of products in accordance with manufacturers' instructions (e.g., dilution, storage, shelf-life, contact time)?

- ◯ My facility performs both audits and feedback for cleaning and disinfection procedures
- ◯ My facility only performs audits for cleaning and disinfection procedures
- ◯ My facility does not perform audits or feedback
- ◯ Not Sure

If yes, what additional resources do you need to strengthen your audit and feedback program for cleaning and disinfection procedures? (Select all that apply)

- ☐ Educational resources to train additional personnel to perform audits
- ☐ Educational resources to train additional personnel on how to provide and receive feedback
- ☐ Standardized cleaning and disinfection procedure audit tool (template or mobile app to assist audits)
- ☐ A tool or database for storing audit and feedback data
- ☐ Directive from accrediting/regulatory group (e.g., CMS)
- ☐ Dedicated FTE for performing audits
- ☐ Leadership support/expectation for audits and feedback to occur
- ☐ Other
- ☐ We have a well-designed and implemented program, no improvements are needed

If Other, please explain

____

If no, what resources do you need to develop an audit and feedback program for cleaning and disinfection procedures? (Select all that apply)

☐ Educational resource to train personnel to perform audits
☐ Educational resource to train personnel on how to provide and receive feedback
☐ Standardized cleaning and disinfection procedure audit tool (template or mobile app to assist audits)
☐ A tool or database for storing audit and feedback data
☐ Dedicated FTE for performing audits
☐ Leadership support/expectation for audits and feedback to occur
☐ Other

If Other, please explain

____

---

19) Does your facility perform audits and feedback on adherence to reprocessing procedures for semi-critical devices, that is, procedures for high-level disinfection (HLD)? NOTE: Examples of devices you might perform HLD for include: endoscopes, speculums, ultrasound probes, or devices that can't undergo heat sterilization.

◯ My facility performs both audits and feedback for reprocessing semi-critical devices
◯ My facility only performs audits for reprocessing semi-critical devices
◯ My facility does not perform audits or feedback for reprocessing semi-critical devices
◯ Not Sure

If yes, what additional resources do you need to strengthen your audit and feedback program for reprocessing semi-critical? (Select all that apply)

☐ Educational resources to train additional personnel to perform audits
☐ Educational resources to train additional personnel on how to provide and receive feedback
☐ Standardized audit tool for reprocessing semi-critical devices (template or mobile app to assist audits)
☐ A tool or database for storing audit and feedback data
☐ Directive from accrediting/regulatory group (e.g. CMS)
☐ Dedicated FTE for performing audits
☐ Leadership support/expectation for audits and feedback to occur
☐ Other
☐ We have a well-designed and implemented program, no improvements are needed

---

If other, please explain

____

If no, what resources do you need to develop an audit and feedback program for reprocessing semi-critical devices? (Select all that apply)

☐ Educational resource to train personnel to perform audits
☐ Educational resource to train personnel on how to provide and receive feedback
☐ Standardized audit tool for reprocessing semi-critical devices (template or mobile app to assist audits)
☐ A tool or database for storing audit and feedback data
☐ Directive from accrediting/regulatory group (e.g. CMS)
☐ Dedicated FTE for performing audits
☐ Leadership support/expectation for audits and feedback to occur
☐ Other
☐ We do not reprocess any semi-critical devices (do not perform HLD)

If other, please explain

____

20) Does your facility perform audits and feedback on adherence to reprocessing procedures for critical devices - that is, procedures for sterilization (e.g., surgical instruments)?

◯ My facility performs both audits and feedback for reprocessing critical devices
◯ My facility only performs audits for reprocessing critical device
◯ My facility does not perform audits or feedback for reprocessing critical devices
◯ Not Sure

If yes, what additional resources do you need to strengthen your audit and feedback program for reprocessing critical devices? (Select all that apply)

☐ Educational resources to train additional personnel to perform audits
☐ Educational resources to train additional personnel on how to provide and receive feedback
☐ Standardized audit tool for reprocessing critical devices (template or mobile app to assist audits)
☐ A tool or database for storing audit and feedback data
☐ Directive from accrediting/regulatory group (e.g. CMS)
☐ Dedicated FTE for performing audits
☐ Leadership support/expectation for audits and feedback to occur
☐ Other
☐ We have a well-designed and implemented program, no improvements are needed

If other, please explain

____

If no, what resources do you need to develop an audit and feedback program for reprocessing critical devices? (Select all that apply)

☐ Educational resource to train personnel to perform audits
☐ Educational resource to train personnel on how provide and receive feedback
☐ Standardized audit tool for reprocessing critical devices (template or mobile app to assist audits)
☐ Educational resource to train personnel on how to receive feedback
☐ A tool or database for storing audit and feedback data
☐ Directive from accrediting/regulatory group (e.g. CMS)
☐ Dedicated FTE for performing audits
☐ Leadership support/expectation for audits and feedback to occur
☐ Other
☐ Do not reprocess any critical devices (do not perform sterilization)

If other, please explain

____

21) Does your facility perform audits and feedback on adherence to recommended infection control (IC) practices for surgical site infection (SSI) prevention? NOTE: IP practices include, but are not limited to preoperative surgical scrub/hand hygiene, appropriate use of surgical drapes, adherence to aseptic technique and maintaining a sterile field.

◯ My facility performs both audits and feedback for IC practices related to SSI prevention
◯ My facility only performs audits for IC practices related to SSI prevention
◯ My facility does not perform audits or feedback for IC practices related to SSI prevention
◯ Not Sure

If yes, what additional resources do you need to strengthen your audit and feedback program for IC practices related to SSI prevention? (Select all that apply)

- ☐ Educational resources to train additional personnel to perform audits
- ☐ Educational resources to train additional personnel on how to provide and receive feedback
- ☐ Standardized audit tool to audit IC practices related to SSI prevention (template or mobile app to assist audits)
- ☐ A tool or database for storing audit and feedback data
- ☐ Dedicated FTE for performing audits
- ☐ Leadership support/expectation for audits and feedback to occur
- ☐ Other
- ☐ We have a well-designed and implemented program, no improvement needed

If other, please explain

____

If no, what resources do you need to develop and provide an audit and feedback program for IC practices related to SSI prevention? (Select all that apply)

- ☐ Educational resource to train personnel to perform audits
- ☐ Educational resource to train personnel on how to provide and receive feedback
- ☐ Standardized audit tool for IP practices related to SSI prevention (template or mobile app to assist audits)
- ☐ A tool or database for storing audit and feedback data
- ☐ Dedicated FTE for performing audits
- ☐ Leadership support/expectation for audits and feedback to occur
- ☐ Other
- ☐ We do not perform surgical procedures

If other, please explain

____

22) Does your facility perform audits and feedback on adherence to recommended infection control practices for C. difficile infection (CDI) prevention?

- ◯ My facility performs both audits and feedback for IC practices related to CDI prevention
- ◯ My facility only performs audits for IC practices related to CDI prevention
- ◯ My facility does not perform audits or feedback for IC practices related to CDI prevention
- ◯ Not Sure

If yes, what additional resources do you need to strengthen your audit and feedback program for CDI prevention practices? (Select all that apply)

- ☐ Educational resources for training additional personnel (e.g., webinar, videos, slide sets, etc.)
- ☐ Educational supplies and equipment
- ☐ Post-educational assessment tools to verify knowledge, skills, and competence
- ☐ On-site or on-staff personnel with adequate subject matter expertise
- ☐ Dedicated FTE for a formal educator
- ☐ Time allotted during employee orientation/onboarding for this topic
- ☐ Annual education, competency verification, and documentation devoted to this topic
- ☐ Directive from accrediting/regulatory group (e.g., CMS)
- ☐ Other
- ☐ We have a well-designed and implemented program, no improvements are needed

If other, please explain

____

If no, what resources do you need to develop and provide an audit and feedback program for IC practices related to CDI prevention? (Select all that apply)

- ☐ Educational resource to train personnel to perform audits
- ☐ Educational resource to train personnel on how to provide and receive feedback
- ☐ Standardized audit tool for IP practices related to SSI prevention (template or mobile app to assist audits)
- ☐ A tool or database for storing audit and feedback data
- ☐ Dedicated FTE for performing audits
- ☐ Leadership support/expectation for audits and feedback to occur
- ☐ Other

If other, please explain

____

Section III--The following questions pertain to the identification and isolation of persons with a serious communicable disease or high-risk pathogen.
Use the definitions below when answering the following questions:
Serious communicable disease (SCD): Pathogens that may require the use of an airborne-infection isolation (AII) room (e.g., MERS, SARS, COVID-19, Ebola)
High Risk Pathogens: Pathogens with potential for high-contact spread (e.g., Acinetobacter, Candida auris, Carbapenem-resistant Enterobacteriaceae (CRE), and Klebsiella Pneumoniae Enterobacteriaceae (KPE)).

Person Under Investigation (PUI): A symptomatic or asymptomatic individual who knowingly or unknowingly has been exposed to a serious communicable disease and/or a high-risk pathogen.

---

1) Does your facility have a system in place that includes travel and symptom screening at all points of entry that allows for rapid identification of a serious communicable disease (SCD) or high-risk pathogen?

◯ Yes ◯ No ◯ Not Sure

If yes, what additional resources does your facility need to improve travel and symptom screening at all points of entry for a SCD or high-risk pathogen? (Select all that apply)

- ☐ Template for appropriate symptom and travel screening build for EMR
- ☐ Template for appropriate symptom and travel screening questions in hard copy
- ☐ Access to current database with current outbreaks of SCD by location
- ☐ Training for personnel who participate in the intake process for patients at all points of entry (access services, triage, RN, MD)
- ☐ Other
- ☐ Our system for travel and symptom screening is well-developed and implemented, not improvements are necessary

---

If Other, please explain

____

If no, what resources does your facility need to be able to develop and implement travel and symptom screening at all points of entry for a SCD or high-risk pathogen? (Select all that apply)

- ☐ Template for appropriate symptom and travel screening build for EMR
- ☐ Template for appropriate symptom and travel screening questions in hard copy
- ☐ Access to current database with current outbreaks of SCD by location
- ☐ Training for personnel who participate in the intake process for patients at all points of entry (access services, triage, RN, MD)
- ☐ Other

If Other, please explain

____

2) Does your facility have a process in place to guide staff on next steps to take once a potential person under investigation (PUI) for a serious communicable disease (SCD) is identified with the use of symptom and travel screening?

◯ Yes ◯ No ◯ Not Sure

If yes, what additional resources does your facility need to improve your process to guide staff on next steps to take once a potential PUI for an SCD is identified during the symptom and travel screening process? (Select all that apply)

☐ State level resource-person, contact person or hotline number
☐ Standard approach guidance (e.g., guideline, protocol template)
☐ Resources for staff training (e.g., slides, on-line course, webinar)
☐ Tools for ongoing readiness (e.g., pocket cards, app, posters, collaborative workgroup, annual readiness verification updates and reminders, webinars)
☐ Designated FTE/protected work time for ongoing readiness
☐ Other
☐ Our system for guiding staff is well-developed and implemented, no improvements are necessary

If Other, please explain

____

If no, what resources does your facility need to improve your process to guide staff on next steps to take once a potential PUI for an SCD is identified during the symptom and travel screening process? (Select all that apply)

☐ State level resource-person, contact person or hotline number
☐ Stable workforce (e.g., mitigation strategies for staff turnover)
☐ Standard approach guidance (e.g., Guideline, protocol template)
☐ Resources for staff training (e.g., slides, on-line course, webinar)
☐ Knowledge assessment tool for post-educational assessment (e.g., quiz forms/responses, checklist, tip-sheets)
☐ Tools for ongoing readiness (e.g., pocket cards, app, posters, collaborative workgroup, annual readiness verification updates and reminders, webinars)
☐ Designated FTE/ Protected work time for ongoing readiness
☐ Other

If Other, please explain

____

3) Does your facility have a process in place to guide staff on next steps to take once a potential person under investigation (PUI) for a high-risk pathogen is identified with the use of symptom and travel screening?

◯ Yes ◯ No ◯ Not Sure

If yes, what additional resources does your facility need to improve your process to guide staff on next steps to take once a potential PUI for a high-risk pathogen is identified during the symptom and travel screening process? (Select all that apply)

☐ State level resource-person, contact person or hotline number
☐ Standard approach guidance (e.g., guideline, protocol template)
☐ Resources for staff training (e.g., slides, on-line course, webinar)
☐ Tools for ongoing readiness (e.g., pocket cards, app, posters, collaborative workgroup, annual readiness verification updates and reminders, webinars)
☐ Designated FTE/protected work time for ongoing readiness
☐ Other
☐ Our system for guiding staff is well-developed and implemented, no improvements are necessary

If Other, please explain

____

If no, what resources does your facility need to improve your process to guide staff on next steps to take once a potential PUI for a high-risk pathogen is identified during the symptom and travel screening process? (Select all that apply)

☐ State level resource-person, contact person or hotline number
☐ Stable workforce (e.g., mitigation strategies for staff turnover)
☐ Standard approach guidance (e.g., Guideline, protocol template)
☐ Resources for staff training (e.g., slides, on-line course, webinar)
☐ Knowledge assessment tool for post-educational assessment (e.g., quiz forms/responses, checklist, tip-sheets)
☐ Tools for ongoing readiness (e.g., pocket cards, app, posters, collaborative workgroup, annual readiness verification updates and reminders, webinars)
☐ Designated FTE/ Protected work time for ongoing readiness
☐ Other

If Other, please explain

____

---

4) Does your facility have a system in place for early detection and rapid isolation of infectious patients identified during their hospital stay?

○ Yes ○ No ○ Not Sure

If yes, what additional resources do you need to improve early detection and rapid isolation of infectious patients identified during their hospital stay? (Select all that apply)

☐ A Lab alert system with ability to notify patient-care staff in real time of suspected/confirmed infections
☐ Electronic medical record ability to flag patients for infections and isolation needs
☐ Leadership support for improving admission procedure/process
☐ Financial support to improve ability of electronic medical record
☐ FTE support for department managing this new system
☐ Training of triage staff
☐ Other
☐ Our system for detection and isolation is well-developed and implemented, no improvements are necessary

---

If Other, please explain

____

If no, what resources do you need to develop a system for early detection and rapid isolation of infectious patients identified during their hospital stay? (Select all that apply)

☐ A Lab alert system with ability to notify patient-care staff in real time of suspected/confirmed infections
☐ Electronic medical record ability to flag patients for infections and isolation needs
☐ Leadership support for improving admission procedure/process
☐ Financial support to improve ability of electronic medical record
☐ FTE support for department managing this new system
☐ Training of triage staff
☐ Other

If Other, please explain

____

5) Does your facility have an airborne-infection isolation room (AIIR) in which to rapidly isolate a PUI who requires additional evaluation?

◯ Yes ◯ No ◯ Not Sure

How many AII rooms?

____

If yes, what additional resources does your facility need to improve your AIIR capabilities? (Select all that apply)

☐ Funding to construct or modify for AII room
☐ Resources to modify and existing non-AII room
☐ National directive or requirement
☐ Training for facilities management staff
☐ Tools for ongoing readiness of facilities management staff (e.g., pocket cards, app, posters, collaborative workgroup, annual readiness verification updates and reminders, webinars)
☐ Access to certification experts for service/validation of air pressure differential testing and balancing
☐ Stable workforce (e.g., mitigation strategies for staff turnover)
☐ Designated FTE/ Protected work time for ongoing readiness
☐ Other
☐ Our system for maintaining our AII rooms is well-developed and implemented, no improvements are necessary

If Other, please explain

____

If no, what resources does your facility need to improve your AIIR capabilities? (Select all that apply)

☐ Funding to construct or modify for AII room
☐ Resources to modify and existing non-AII room
☐ National directive or requirement
☐ Training for facilities management staff
☐ Tools for ongoing readiness of facilities management staff (e.g., pocket cards, app, posters, collaborative workgroup, annual readiness verification updates and reminders, webinars)
☐ Greater access to certification experts for service/validation of air pressure differential testing and balancing
☐ Stable workforce (e.g., mitigation strategies for staff turnover)
☐ Designated FTE/ Protected work time for ongoing readiness
☐ Other

If Other, please explain

____

6) Has your facility developed a process to guide the selection and use of PPE for a PUI suspected/confirmed with a serious communicable disease based on the transmission of the pathogen?

◯ Yes ◯ No ◯ Not Sure

If yes, what additional resources does your facility need to improve your process to guide selection and use of PPE for patients/persons with a SCD based on transmission of the pathogen? (Select all that apply)

☐ Regulatory directive
☐ Administrative buy-in
☐ National standard resource to guide practice (e.g., interactive web-based tool, app, database)
☐ Resources for staff training (e.g., slides, on-line course, webinar)
☐ Resources for verification of staff competency (e.g., quiz forms/responses, table-top drill exercises)
☐ Designated FTE/ Protected work time for ongoing readiness
☐ Other
☐ Our guidance process for staff selecting appropriate PPE is well-developed and implemented, no improvements are necessary

If Other, please explain

____

If no, what does your facility need to be successful in developing processes to guide selection and use of PPE for patients/persons with a SCD based on transmission of the pathogen? (Select all that apply)

☐ Regulatory directive
☐ Administrative buy-in
☐ National standard resource to guide practice (e.g., interactive web-based tool, app, database)
☐ Resources for staff training (e.g., slides, on-line course, webinar)
☐ Resources for verification of staff competency (e.g., quiz forms/responses, table-top drill exercises)
☐ Designated FTE/ Protected work time for ongoing readiness
☐ Other

If Other, please explain

____

7) Has your facility developed a process to guide the selection and use of PPE for a PUI suspected/confirmed with a high-risk pathogen based on the transmission of the pathogen?

◯ Yes ◯ No ◯ Not Sure

If yes, what additional resources does your facility need to improve your process to guide selection and use of PPE for patients/persons with a high-risk pathogen based on transmission of the pathogen? (Select all that apply)

☐ Regulatory directive
☐ Administrative buy-in
☐ National standard resource to guide practice (e.g., interactive web-based tool, app, database)
☐ Resources for staff training (e.g., slides, on-line course, webinar)
☐ Resources for verification of staff competency (e.g., quiz forms/responses, table-top drill exercises)
☐ Designated FTE/ Protected work time for ongoing readiness
☐ Other
☐ Our guidance process for staff selecting appropriate PPE is well-developed and implemented, no improvements are necessary

If Other, please explain

____

If no, what does your facility need to be successful in developing processes to guide selection and use of PPE for patients/persons with a high-risk pathogen based on transmission of the pathogen? (Select all that apply)

- ☐ Regulatory directive
- ☐ Administrative buy-in
- ☐ National standard resource to guide practice (e.g., interactive web-based tool, app, database)
- ☐ Resources for staff training (e.g., slides, on-line course, webinar)
- ☐ Resources for verification of staff competency (e.g., quiz forms/responses, table-top drill exercises)
- ☐ Designated FTE/ Protected work time for ongoing readiness
- ☐ Other

If Other, please explain

____

8) Have personnel that are anticipated to provide care for a PUI received competency-based training on the donning and doffing of PPE that would be used for a serious communicable disease?

◯ Yes ◯ No ◯ Not Sure

If yes, what additional resources does your facility need to ensure competency-based training on the donning and doffing of PPE that would be used for a serious communicable disease? (Select all that apply)

- ☐ Additional supplies and equipment used in donning and doffing PPE for training purposes
- ☐ Reliable supply chain
- ☐ Regulatory directive
- ☐ Administrative buy-in
- ☐ Knowledge assessment tool for post-educational assessment
- ☐ Skills verification checklist
- ☐ On-site or on-staff personnel with adequate subject matter expertise
- ☐ Dedicated FTE for a formal educator
- ☐ Time allotted during employee orientation for this topic
- ☐ Annual staff education time devoted to this topic
- ☐ Designated FTE/ Protected work time for ongoing readiness
- ☐ Stable workforce (e.g., mitigation strategies for staff turnover)
- ☐ Other
- ☐ Our system for PPE competency for donning and doffing is well-developed and implemented, no improvements are necessary

If Other, please explain

____

If no, what does your facility need to be successful in providing competency-based training on the donning and doffing of PPE that would be used for a serious communicable disease? (Select all that apply)

- ☐ Additional supplies and equipment used in donning and doffing PPE for training purposes
- ☐ Reliable supply chain
- ☐ Regulatory directive
- ☐ Administrative buy-in
- ☐ Knowledge assessment tool for post-educational assessment
- ☐ Skills verification checklist
- ☐ On-site or on-staff personnel with adequate subject matter expertise
- ☐ Dedicated FTE for a formal educator
- ☐ Time allotted during employee orientation for this topic
- ☐ Annual staff education time devoted to this topic
- ☐ Designated FTE/ Protected work time for ongoing readiness
- ☐ Stable workforce (e.g., mitigation strategies for staff turnover)
- ☐ Other

If Other, please explain

____

---

9) Does your facility have personal protective respiratory equipment available for the care of patients in airborne infection isolation (AII), such as N95 respirators and/or PAPRs?

◯ Yes ◯ No ◯ Not Sure

If yes, what additional resources does your facility need to improve personal protective respiratory equipment availability for the care of patients in AII? (Select all that apply)

- ☐ Stable workforce (e.g., mitigation strategies for staff turnover)
- ☐ Additional supplies and equipment types
- ☐ Tracking tools for equipment maintenance
- ☐ Reliable supply chain
- ☐ Financial assistance to ensure adequate par-level supplies
- ☐ Regulatory directive
- ☐ Administrative buy-in
- ☐ Access to ongoing equipment readiness guidance (e.g., pocket cards, app, posters, collaborative workgroup, annual readiness verification updates and reminders, webinars)
- ☐ Designated FTE/ Protected work time for ongoing readiness
- ☐ Other
- ☐ Our system for respiratory equipment availability and use is well-developed and implemented, no improvements are necessary

---

If Other, please explain

____

If no, what does your facility need to be successful in ensuring that personal protective respiratory equipment is available for staff use during care of patients in AII rooms? (Select all that apply)

☐ Stable workforce (e.g., mitigation strategies for staff turnover)
☐ Additional supplies and equipment types
☐ Tracking tools for equipment maintenance
☐ Reliable supply chain
☐ Financial assistance to ensure adequate par-level supplies
☐ Regulatory directive
☐ Administrative buy-in
☐ Access to ongoing equipment readiness guidance (e.g., pocket cards, app, posters, collaborative workgroup, annual readiness verification updates and reminders, webinars)
☐ Designated FTE/ Protected work time for ongoing readiness
☐ Other

If Other, please explain

____

---

10) Does your facility have a plan in place to process Category A hazardous substance waste that is generated during the care of a PUI or person confirmed to have serious communicable disease (e.g., waste generated with a suspected/actual Ebola patient)?

◯ Yes ◯ No ◯ Not Sure

If yes, what additional resources does your facility need to improve you plan to process Category A hazardous substance waste that is generated during the care of a PUI or person confirmed to have a SCD? (Select all that apply)

☐ Risk Assessment Template
☐ Mitigation toolkit
☐ Additional supplies and equipment types
☐ Stable workforce (e.g., mitigation strategies for staff turnover)
☐ Reliable supply chain
☐ Financial assistance to ensure adequate par-level supplies
☐ Regulatory directive
☐ Administrative buy-in
☐ Access to ongoing equipment readiness guidance (e.g., pocket cards, app, posters, collaborative workgroup, annual readiness verification updates and reminders, webinars)
☐ Other
☐ Our process for handling Category A waste is well-developed and implemented, no improvements are necessary

---

If Other, please explain

____

If no, what does your facility need to be successful to have a plan in place to process Category A hazardous substance waste generated during care of a PUI or confirmed person with a SCD? (Select all that apply)

☐ Risk Assessment Template
☐ Mitigation toolkit
☐ Additional supplies and equipment types
☐ Stable workforce (e.g., mitigation strategies for staff turnover)
☐ Reliable supply chain
☐ Financial assistance to ensure adequate par-level supplies
☐ Regulatory directive
☐ Administrative buy-in
☐ Access to ongoing equipment readiness guidance (e.g., pocket cards, app, posters, collaborative workgroup, annual readiness verification updates and reminders, webinars)
☐ Other

If Other, please explain

____

11) Does your facility have a process in place to coordinate and support the transfer of a PUI or person confirmed to have serious communicable disease or high-risk pathogen to another facility based on jurisdictional guidance?

◯ Yes ◯ No ◯ Not Sure

If yes, what additional resources does your facility need to improve your process to coordinate and support the transfer of a PUI or person confirmed to have a SCD or high-risk pathogen to another facility? (Select all that apply)

☐ Stable workforce (e.g., mitigation strategies for staff turnover)
☐ Access to ongoing readiness guidance (e.g., pocket cards, app, posters, collaborative workgroup, annual readiness verification updates and reminders, webinars)
☐ Regulatory directive
☐ Administrative buy-in
☐ On-site or on-staff personnel with adequate subject matter expertise
☐ Annual staff education time devoted to this topic
☐ Direct communication network with transfer-facility
☐ Other
☐ Our process for transferring a PUI or confirmed SCD patient is well-developed and implemented, no improvements are necessary

If Other, please explain

____

If no, what does your facility need to be successful in putting a process in place to coordinate and support the transfer of a PUI or person confirmed to have a SCD or highh-risk pathogen to another facility? (Select all that apply)

☐ Stable workforce (e.g., mitigation strategies for staff turnover)
☐ Access to ongoing readiness guidance (e.g., pocket cards, app, posters, collaborative workgroup, annual readiness verification updates and reminders, webinars)
☐ Regulatory directive
☐ Administrative buy-in
☐ On-site or on-staff personnel with adequate subject matter expertise
☐ Annual staff education time devoted to this topic
☐ Direct communication network with transfer-facility
☐ Other

If Other, please explain

____

12) Does your facility have a system in place for asking/identifying isolation status and needs of patients transferring to your facility from another facility?

◯ Yes ◯ No ◯ Not Sure

If yes, what additional resources do you need to improve/strengthen your system/process for identifying isolation status and needs of patients transferring to your facility from another facility? (Select all that apply)

☐ Written intake procedure/process that includes questions on infections and isolation needs
☐ Education for additional personnel who admit patients on the written procedure/process
☐ Electronic medical record ability to flag patients for infections and isolation needs
☐ Leadership support for improving admission procedure/process
☐ Financial support to improve ability of electronic medical record
☐ Directive from accrediting/regulatory group (e.g. CMS or the state)
☐ Standardized transfer form which includes isolation needs
☐ Other

If Other, please explain

____

If no, what resources do you need to be successful in implementing of a system, which would identify isolation status and needs of patients transferring to your facility from another facility?(Select all that apply)

☐ Written intake procedure/process that includes questions on infections and isolation needs
☐ Education for additional personnel who admit patients on the written procedure/process
☐ Electronic medical record ability to flag patients for infections and isolation needs
☐ Leadership support for improving admission procedure/process
☐ Financial support to improve ability of electronic medical record
☐ Directive from accrediting/regulatory group (e.g. CMS or the state)
☐ Standardized transfer form which includes isolation needs
☐ Other

If Other, please explain

____

13) Does your facility have a protocol/plan in place to notify jurisdictional public health authorities when a PUI is identified?

◯ Yes ◯ No ◯ Not Sure

If yes, what additional resources does your facility need to improve the system in place to notify jurisdictional public health authorities when a PUI is identified? (Select all that apply)

☐ Stable workforce (e.g., mitigation strategies for staff turnover)
☐ Access to ongoing readiness guidance (e.g., pocket cards, app, posters, collaborative workgroup, annual readiness verification updates and reminders, webinars)
☐ Regulatory directive
☐ Administrative buy-in
☐ On-site or on-staff personnel with adequate subject matter expertise
☐ Annual staff education time devoted to this topic
☐ Direct communication network with jurisdictional public health authorities
☐ Other

If Other, please explain

____

If no, what does your facility need to be successful in having a system in place to notify jurisdictional public health authorities when a PUI is identified? (Select all that apply)

☐ Stable workforce (e.g., mitigation strategies for staff turnover)
☐ Access to ongoing readiness guidance (e.g., pocket cards, app, posters, collaborative workgroup, annual readiness verification updates and reminders, webinars)
☐ Regulatory directive
☐ Administrative buy-in
☐ On-site or on-staff personnel with adequate subject matter expertise
☐ Annual staff education time devoted to this topic
☐ Direct communication network with jurisdictional public health authorities
☐ Other

If Other, please explain

____

14) Has your facility conducted a risk assessment for the laboratory, identifying what tests can safely be offered in order to provide appropriate clinical care for a PUI?

◯ Yes ◯ No ◯ Not Sure

If yes, what additional resources does your facility need to improve your process for conducting a risk assessment of the laboratory and identifying what lab tests can safely be offered in order to provide appropriate clinical care for a PUI? (Select all that apply)

☐ Risk Assessment Template
☐ Mitigation toolkit
☐ Additional supplies and equipment types
☐ Stable workforce (e.g., mitigation strategies for staff turnover)
☐ Reliable supply chain
☐ Financial assistance to ensure adequate par-level supplies
☐ Regulatory directive
☐ Administrative buy-in
☐ Access to ongoing equipment readiness guidance (e.g., pocket cards, app, posters, collaborative workgroup, annual readiness verification updates and reminders, webinars)
☐ Other
☐ Our laboratory risk assessment has not identified any issues, no improvements are necessary

If Other, please explain

____

If no, what does your facility need to be successful in conducting a risk assessment of the lab and identification of what lab tests can safely be offered in order to provide appropriate clinical care for a PUI? (Select all that apply)

☐ Risk Assessment Template
☐ Mitigation toolkit
☐ Additional supplies and equipment types
☐ Stable workforce (e.g., mitigation strategies for staff turnover)
☐ Reliable supply chain
☐ Financial assistance to ensure adequate par-level supplies
☐ Regulatory directive
☐ Administrative buy-in
☐ Access to ongoing equipment readiness guidance (e.g., pocket cards, app, posters, collaborative workgroup, annual readiness verification updates and reminders, webinars)
☐ Other

If Other, please explain

____
